# Synthesis and Spectroscopic Characterization of Benzimidazole-Derived Schiff Base: Investigation of Optical Properties, DNA Binding, DFT, and Molecular Docking

**DOI:** 10.3390/molecules31091513

**Published:** 2026-05-02

**Authors:** Ahmed N. Alhakimi, Sadeq M. Al-Hazmy, Ibrahim A. Alhagri, Sabri Messaoudi, Ahmed Kaid Alantry, Tahani Alresheedi

**Affiliations:** 1 Department of Chemistry, College of Science, Qassim University, Buraidah 51452, Saudi Arabia; a.alhakimi@qu.edu.sa (A.N.A.); s.alhzmy@qu.edu.sa (S.M.A.-H.); eb.ahmed@qu.edu.sa (I.A.A.); s.messaoudi@qu.edu.sa (S.M.); 2 Department of Physiology, College of Medicine, Qassim University, Buraydah 51452, Saudi Arabia; ak.alantry@qu.edu.sa

**Keywords:** synthesis, benzimidazole, Schiff base, optical properties, DNA, docking, UV-Vis and fluorescence spectroscopy

## Abstract

This study reports the synthesis and characterization of a novel benzimidazole-derived Schiff base (BIMPB) via the condensation of (1H-benzo[d]imidazol-2-yl)methanamine with 1-phenylbutane-1,3-dione. The structure was confirmed using ^1^H-NMR, ^13^C-NMR and FT-IR spectroscopy. Photophysical properties were extensively evaluated, revealing a strong S_0_ → S_2_ transition at 212 nm and fluorescence emission peaks at 396 and 410 nm, corresponding to π → π* and n → π* transitions. BIMPB demonstrated significant sensitivity to pH variations, exhibiting blue shifts of 11–23 nm across different environments. Furthermore, the compound acts as a fluorescent chemosensor for Cu^2+^ and Ca^2+^ ions, where coordination leads to a substantial reduction in fluorescence intensity accompanied by a distinct blue shift. The interaction between BIMPB and DNA was investigated using UV-Vis and fluorescence titration. The results showed a hypochromic effect and a minor shift in the absorption peak from 342 nm to 340 nm, suggesting a binding mechanism dominated by intercalation or electrostatic interactions. A high binding constant (K_b_ = 2.1 × 10^5^ M^−1^) and a fluorescence quenching efficiency of 58.9% confirm the formation of a stable complex. Stern–Volmer analysis indicated a static quenching mechanism. These experimental findings, supported by molecular docking studies (binding energy = −8.3 kcal/mol), highlight the potential of BIMPB as a sensitive molecular probe for DNA-targeting and chemical sensing applications.

## 1. Introduction

Schiff bases, which are distinguished by the imine (-C=N-) functional group, have been extensively studied in biomedical sciences due to their wide range of biological activities, including antibacterial, antioxidant, and anticancer properties [1,2]. Their simple synthesis, structural diversity, and simplicity of modification endow them with promising therapeutic development potential [3]. Schiff bases produced from heterocyclic systems have demonstrated increased biological activity as a result of synergistic effects between imine functionality and heterocyclic moiety [4]. Benzimidazole has received a lot of interest as a pharmacophore because of its structural resemblance to purine bases, which allows for easier interaction with nucleic acids and proteins [5]. The Schiff bases formed between benzimidazole derivatives and β-diketones offer unique physicochemical characteristics, including enhanced conjugation, tunable electronic properties, and potential biological activity. These compounds have been widely investigated for their antimicrobial, anticancer, antioxidant, and catalytic properties, as well as their roles as ligands in metal coordination chemistry. In this context, the present study explores the synthesis, characterization, and potential applications of such Schiff bases, highlighting their significance in both fundamental and applied research [6,7,8]. Benzimidazole, a fused heterocyclic system containing benzene and imidazole moieties, exhibits strong nucleophilic and hydrogen-bonding capabilities, making it a valuable precursor for Schiff base formation. Nitrogen in benzimidazole enhances its coordination ability with metal centers, leading to the development of metal complexes with interesting electronic, catalytic, and biologi

cal properties. Meanwhile, β-diketones are well-known for their enol–keto tautomerism and strong electron-donating properties, further influencing the stability and reactivity of Schiff bases. Schiff bases derived from benzimidazole and β-diketone compounds have gained significant attention due to their diverse structural versatility and wide range of applications in coordination chemistry, medicinal chemistry, and materials science [7,9,10].

Schiff bases and their metal complexes are increasingly recognized for their impressive biological activities against bacteria, fungi, and tumors [11,12]. Recent advancements in the development of chemical sensors have highlighted the potential of Schiff bases [13,14,15], particularly in detecting heavy metals and toxic contaminants. Their unique tautomerization processes induce significant changes in the electronic structure, rendering them suitable for use as anion sensors. The presence of intramolecular hydrogen bonds and the ability for proton transfer further enhance their functionality in sensor applications [16,17].

In our study, we synthesized a novel Schiff base ((E)-3-(((1H-benzo[d]imidazol-2-yl)methyl)imino)-1-phenylbutan-1-one (BIMPB)), which exhibits remarkable sensitivity and selectivity. We employed spectroscopic methods, including UV-vis and photoluminescence analyses, to investigate the absorption and emission properties of this Schiff base across various solvents. Notably, we observed significant variations in extinction coefficients, emphasizing the solvent dependency of the fluorescence absorption spectra [18,19].

Additionally, the exploration of concentration effects on normal-to-tautomer ratios revealed crucial insights into the interactions between substituents, solvents, and fluorescence characteristics. The colorimetric analysis and the study of the influence of solvent viscosity confirmed the enhancement in fluorescence efficiency achieved by reducing non-radiative transitions [20,21].

Numerous investigations have shown that benzimidazole derivatives have anticancer properties, notably through DNA binding, enzyme inhibition, and the disruption of cellular signaling pathways [22,23]. The addition of the benzimidazole unit to Schiff bases has been demonstrated to increase their biological activity by strengthening DNA binding interactions and boosting cytotoxicity against cancer cells [24,25].

DNA, which is a main target for many chemotherapeutic medicines, has been intensively researched to better understand possible pharmacological mechanisms [26]. Small molecules can interact with DNA through a variety of mechanisms, including intercalation, groove binding, and electrostatic interactions [27,28]. UV-Vis absorption and fluorescence spectroscopy have evolved as effective methods for studying these interactions. UV-Vis tests often show variations in absorption maxima (λ_max_) and intensity, such as hypochromic, bathochromic, or hypsochromic shifts, indicating binding events [29]. Similarly, fluorescence spectroscopy gives insight into the binding mechanism via quenching events, which may be distinguished between dynamic and static quenching processes using the Stern–Volmer equation [30,31].

Recent research has shown that several Schiff bases interact with DNA via intercalation, resulting in dramatic changes in both the spectral and biological profiles of the compounds [32,33,34]. For example, various benzimidazole-based Schiff bases were tested for DNA binding affinities, and moderate to high binding constants were found, indicating the creation of persistent drug–DNA complexes [35]. Such interactions are critical not only for understanding the pharmacodynamics of these drugs but also for developing molecules with greater selectivity and efficacy against cancer cells [36,37]. Furthermore, investigations comparing the binding modes of these compounds using both UV-Vis and fluorescence spectroscopic methods have repeatedly shown the relevance of static quenching processes, which are responsible for the creation of ground-state complexes [38].

In addition to DNA binding investigations, current research focuses on the cytotoxicity of benzimidazole-derived Schiff bases. Several studies have linked the DNA binding capacity of these chemicals to their potential to cause apoptosis in cancer cells [39]. Their cytotoxic potential is thought to stem from interference with DNA replication and transcription activities, which results in cell cycle arrest and eventual cell death [40]. Furthermore, structure–activity relationship (SAR) investigations have shed light on how changes to the benzimidazole or imine moieties impact overall biological activity, leading to the rational development of more effective anticancer drugs [41,42].

Despite these advances, achieving the ideal balance of binding affinity, selectivity, and cytotoxicity remains a problem. To enhance treatment results, it is crucial to understand the specific binding processes and stoichiometry of drug–DNA complexes [20]. Recent research using a combination of spectroscopic and computational techniques has shown promise in overcoming these issues by providing a more precise molecular-level knowledge of the interactions [43]. Furthermore, new compounds with enhanced pharmacokinetic characteristics are required to address the constraints associated with drug resistance and toxicity [44,45].

This work describes the synthesis of a new benzimidazole-derived Schiff base (BIMPB) and evaluates its DNA binding properties through UV-Vis and fluorescence spectroscopy. By determining the binding constants and quenching parameters, this study provides a clearer understanding of the compound’s molecular interactions. These findings contribute to the characterization of benzimidazole-based compounds and offer insights that may support the future design of DNA-targeted molecular probes.

## 2. Materials and Methods

### 2.1. Synthesis of (E)-3-(((1H-Benzo[d]imidazol-2-yl)methyl)imino)-1-phenylbutan-1-one (BIMPB)

In this experiment, we used chemicals from Sigma-Aldrich (St. Louis, MO, USA) without analysis to synthesize (E)-3-(((1H-benzo[d]imidazol-2-yl)methyl)imino)-1-phenylbutan-1-one, and the following optimized procedure was followed:

(1H-benzo[d]imidazol-2-yl)methanamine (1.0 mmol) and 1-phenylbutane-1,3-dione (1.0 mmol) were mixed in ethanol (EtOH) (20 mL) as the reaction solvent. The reactants were dissolved in ethanol under constant stirring to ensure homogeneity. The reaction mixture was heated to 80 °C and stirred for three hours, allowing the Schiff base condensation reaction to proceed efficiently. Upon completion, the reaction was cooled to room temperature, leading to the formation of a red precipitate. The solid product was collected via filtration and washed several times with hot ethanol to remove any unreacted starting materials or side products.

**Scheme 1:**
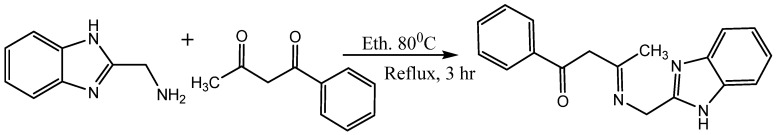
Synthesis of BIMPB compound.

Finally, the purified Schiff base was left to air dry under a vacuum to obtain the final compound in high purity (Scheme 1).

### 2.2. UV-Vis and Fluorescence Spectra

A modified A. J. Lees method, which takes into account the decrease in absorbance at the excitation wavelength as photo-irradiation proceeds, was followed to measure the photochemical quantum yields of BIMPB (ϕ_c_). A Jasco FP-8200 spectrofluorometer (Jasco, Tokyo, Japan) was used to record the steady-state fluorescence spectra with a quartz cuvette of 1 cm path length. The emission spectra were recorded using an emission slit width of 5 nm at a 90° angle relative to the excitation beam, with a xenon lamp as the light source. Depending on the emission wavelength range, fluorescence quantum yields were computed using Equation (1) and calculated using the optically diluted solution relative method with 9,10-diphenyleanthracene solutions. Ferrioxalate actinometry was used to measure light intensity [46].

(1) $$\emptyset_{f}\left(s\right)=\emptyset_{f}\left(r\right)\times \frac{\int I_{s}}{\int I_{r}}\times \frac{A_{r}}{A_{s}}\times \frac{n_{s}^{2}}{n_{r}^{2}}$$

The corrected fluorescence peak area is denoted by the integral denote, *A* refers to the absorbance at the excitation wavelength, and n refers to the solvent’s refractive index. The subscripts r and s indicate the reference and sample, respectively. $\emptyset_{f}$ is the fluorescence quantum yield.

### 2.3. DNA Studies

Sigma provided salmon DNA, which was used without any purifying procedures. To make stock solutions, it was dissolved in deionized water. By measuring UV absorbance at 260 nm and using an extinction value of 6600 M^−1^cm^−1^, the content of DNA was determined [47]. The A260/A280 ratio was used to validate the purity of the sample; it was found to be 1.89, which is over the 1.8 threshold, suggesting high-quality DNA devoid of protein contamination [48]. To maintain appropriate experimental conditions for DNA interaction assays, a 10 mM Tris buffer was made, and its pH was carefully adjusted to 7.4 using hydrochloric acid.

### 2.4. Molecular Docking

The organic compound BIMPB was geometrically optimized using the conjugate gradient method implemented in AMMP software version 3.2.3 [49]. The crystal structure of the B-DNA dodecamer d(CGCGAATTCGCG)_2_ (PDB ID: 1BNA) was downloaded from the Protein Data Bank to serve as the target macromolecule. Molecular docking simulations were performed using AutoDock Vina to investigate the binding interactions between BIMPB and B-DNA [50]. The DNA structure was prepared for docking using AutoDock 4.2 Tools (ADT) [51]. Preparation steps included the removal of water molecules, addition of polar hydrogens, and assignment of Gasteiger partial charges to accurately model electrostatic interactions. A docking grid box was established with dimensions of 60 × 60 × 110 Å and a grid spacing of 0.375 Å, centered at coordinates x = 14.779, y = 20.976, and z = 8.804. This grid configuration ensured the comprehensive coverage of the B-DNA structure, allowing the docking algorithm to explore all potential binding sites without constraints. The resulting binding conformations and intermolecular interactions were visualized and analyzed using BIOVIA Discovery Studio Visualizer [52].

## 3. Results and Discussion

**Scheme 2:**
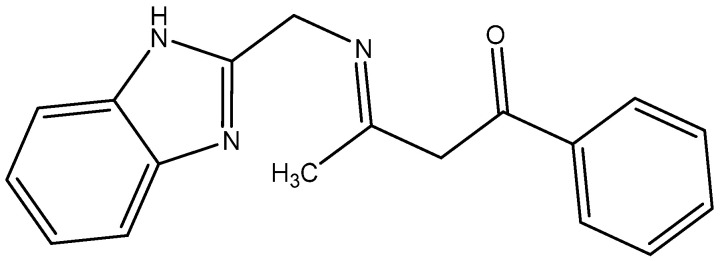
(E)-3-(((1H-benzo[d]imidazol-2-yl)methyl)imino)-1-phenylbutan-1-one (BIMPB).

(E)-3-(((1H-benzo[d]imidazol-2-yl)methyl)imino)-1-phenylbutan-1-one (BIMPB) (Scheme 2) is a Schiff base ligand. The illustrated compound is a heterocyclic Schiff base derivative incorporating a fused benzoimidazole ring system connected through an imine (-CH=N-) linkage to a substituted carbonyl-bearing side chain. The core bicyclic scaffold contains two nitrogen atoms, which endow the molecule with significant electron-donating and hydrogen-bonding capabilities, contributing to its potential biological and coordination properties. Schiff base frameworks of this type are known for their structural versatility, thermal stability, and ability to form stable complexes with transition metals.

The presence of both an imine group and a carbonyl moiety introduce conjugation across the molecular backbone, enhancing the overall electron delocalization and potentially improving reactivity toward nucleophiles or electrophiles. The attached phenyl ring further extends the π-system, which may influence the compound’s optical, electronic, or biological characteristics. Due to these features, molecules of this structural class have attracted considerable interest in medicinal chemistry, coordination chemistry, and materials science, where they frequently exhibit antimicrobial, anticancer, antioxidant, and catalytic activities. Accordingly, the designed structure represents a promising ligand framework for exploring structure–activity relationships and developing new functional materials.

### 3.1. ^1^H and ^13^C NMR Spectroscopy

**Figure 1:**
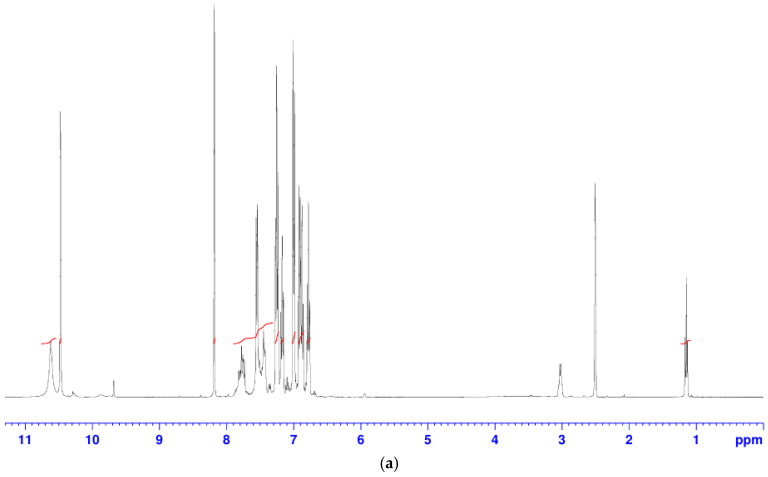

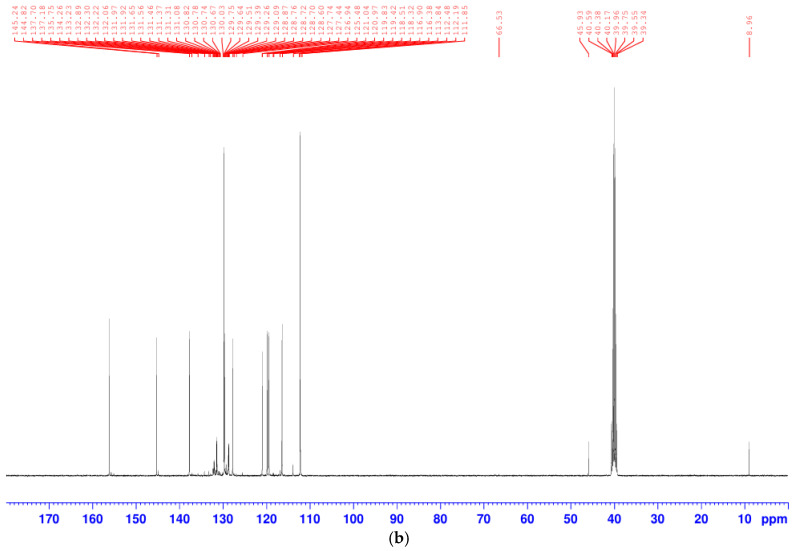
(**a**) The ^1^H-NMR spectra of BIMPB. (**b**) The ^13^C-NMR spectra of BIMPB.

The ^1^H NMR spectrum of the synthesized ligand displays well-resolved signals consistent with its proposed structure. The methyl protons of the benzoylacetone fragment appear as an intense triplet at approximately 2.50 ppm, clearly indicating their coupling with the adjacent methylene group. The aliphatic methylene protons located next to the azomethine (–CH=N–) moiety exhibit a doublet pattern with a noticeable downfield shift relative to typical aliphatic –CH_2_– groups, a shift attributed to the electron-withdrawing effect of the neighboring imine nitrogen. The methylene protons within the diketone backbone resonate at a slightly higher chemical shift compared to the former, further supporting the predominance of the keto tautomeric form over the enol form. The aromatic region shows several multiplets distributed between 7.16 and 8.40 ppm, corresponding to the phenyl ring protons. Minor low-intensity peaks were also observed in this region, likely arising from restricted rotation around the C=N bond or the presence of trace conformers, which is commonly reported for structurally related Schiff bases, as shown in Figure 1a. The ^13^C NMR spectrum further corroborates the structural assignment. Multiple resonances spanning the range of 20–155 ppm were detected. The methyl carbon of the benzoylacetone moiety appears at ~17 ppm with a strong and sharp signal. The methylene carbons adjacent to the azomethine group and the central methylene carbon in benzoylacetone resonate at 48 and 45 ppm, respectively; both signals exhibit high intensity, reflecting their symmetric chemical environments. The imine carbon (C=N) is clearly identified at 145 ppm, while the carbonyl carbon of the benzoylacetone unit resonates at 155 ppm, showing pronounced deshielding due to the strong electron-withdrawing effects of adjacent nitrogen and oxygen atoms. Small additional resonances of very low intensity are observed in the aliphatic and aromatic regions, which may be attributed to minor rotameric forms or keto–enol interconversion, although the dominant species remains unequivocally the keto form. Aromatic carbons appear within the expected region of 115–142 ppm, aligning well with reported values for similar substituted aromatic Schiff bases, as shown in Figure 1b.

Collectively, the NMR data, including chemical shift trends, splitting patterns, and the presence of minor satellite peaks, confirm that the ligand predominantly exists in the keto tautomeric form and validate the proposed molecular structure [53,54].

### 3.2. Infrared Spectroscopy Analysis

**Figure 2:**
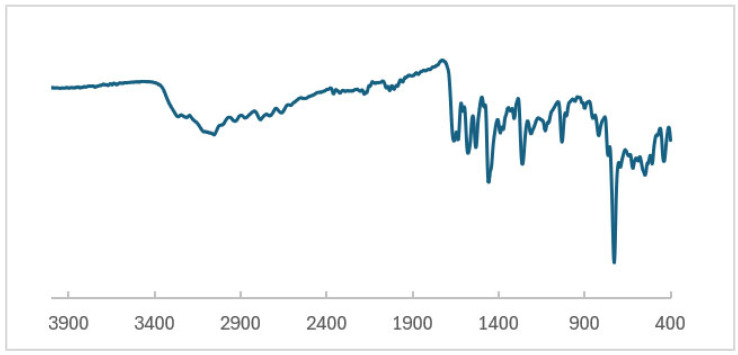
The IR spectrum of BIMPB.

The infrared (IR) spectrum exhibited several characteristic absorption bands, confirming the formation of the Schiff base. Notably, the disappearance of the amine (-NH_2_) stretching band of the benzimidazole precursor, coupled with the emergence of a distinct absorption band at 1610 cm^−1^, is indicative of the formation of the azomethine (-C=N-) functional group through condensation with acetylacetone. Additionally, the spectrum displayed absorption bands around 3100 cm^−1^, corresponding to the N-H stretching vibration of the cyclic nitrogen within the benzimidazole moiety, further supporting the structural integrity of the synthesized compound, as shown in Figure 2 [53].

### 3.3. UV–Visible and Fluorescence Spectra of BIMPB

**Figure 3:**
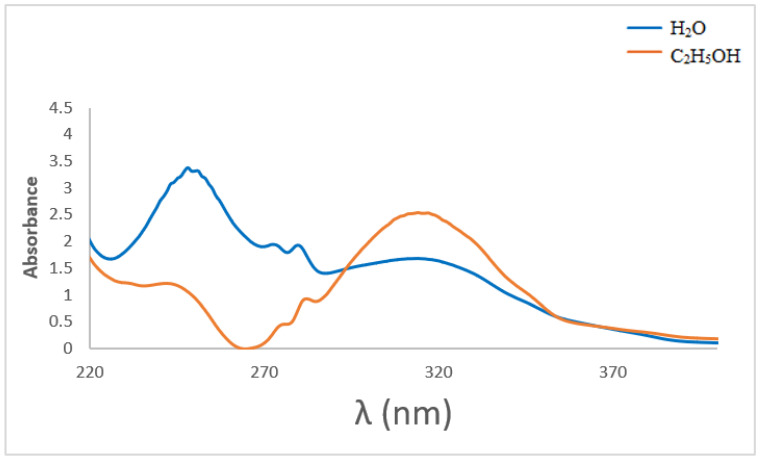
Absorption spectra of 4 × 10^−5^ M BIMPB in ethanol and H_2_O.

The spin-allowed S_0_ → S_2_ transition is largely responsible for the relatively strong absorbance at 212 nm and the peak-like shoulder at 245 nm that BIMPB exhibits in ethanol. As illustrated in Figure 3, the spin-allowed S_0_ → S_1_ transition of the benzoimidazole moiety is related to the lower-intensity band at 317 nm. The spin-allowed S_0_ → S_2_ transition is primarily responsible for BIMPB’s relatively strong absorbance at 212 nm in aqueous solution. The spin-allowed S_0_ → S_1_ transition and hydrogen bonding formed with H_2_O molecules may be responsible for the shoulder-like peak at 304 nm [55].

**Figure 4:**
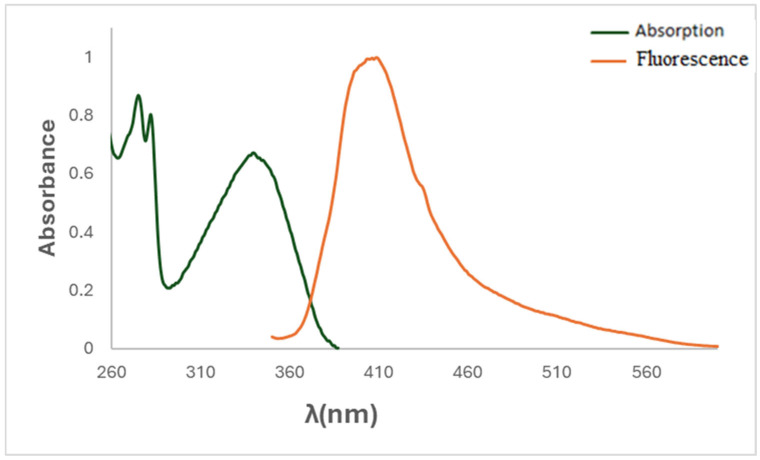
Normalized absorption and fluorescence spectra (λ_ex_ = 345 nm) of 4 × 10^−5^ M BIMPB in CH_2_Cl_2_.

**Figure 5:**
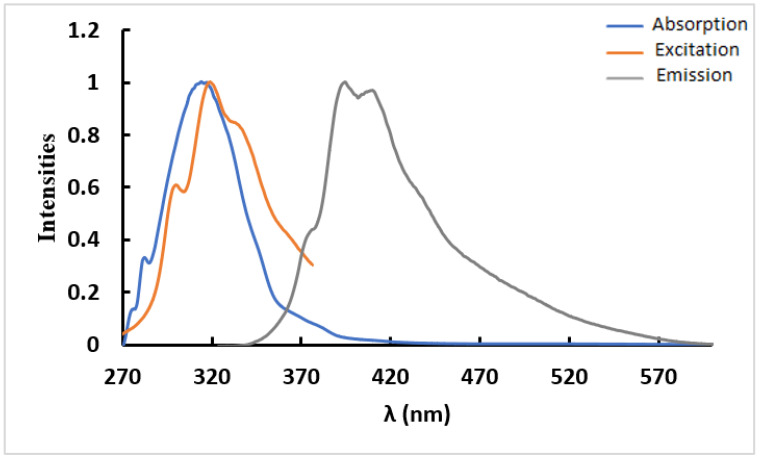
Absorption, fluorescence, λ_ex_ = 314 nm and excitation spectra of BIMPB in ethanol, λ_em max_ = 400 nm.

A substantial Stokes shift of around 1.43 × 10^5^ cm^−1^ for 4 × 10^−5^ M of BIMPB in CH_2_Cl_2_ was observed, due to the initial excited state’s vibrational relaxation to the first electronic excited state’s lowest vibrational state. Figure 4 shows a red-shifted emission that occurs due to the return to a variety of vibrational levels in the ground state. The red shift in the fluorescence maximum wavelength may also be attributed to the development of a highly polar excited state that, immediately after excitation, relaxes the surrounding solvent cage [27]. The substantial Stokes shift exhibited by the dye in Figure 5 is a highly advantageous characteristic, as it minimizes the reabsorption of emitted photons. This property enables efficient fluorescence emission even at elevated concentrations, positioning the material as a strong candidate for applications in optical devices. The subtle structural differences observed between the absorption and emission spectra may arise from the distinct energy levels of the ground and excited states. This suggests that the electronic transitions governing absorption and emission processes are not directly correlated, as depicted in Figure 5. Furthermore, the close alignment between the excitation and absorption spectra underscores the relative purity of the dye under investigation [3,13].

### 3.4. Medium Effect: The Impact of Solvent Polarity on UV–Visible and Fluorescence Characteristics

**Figure 6:**
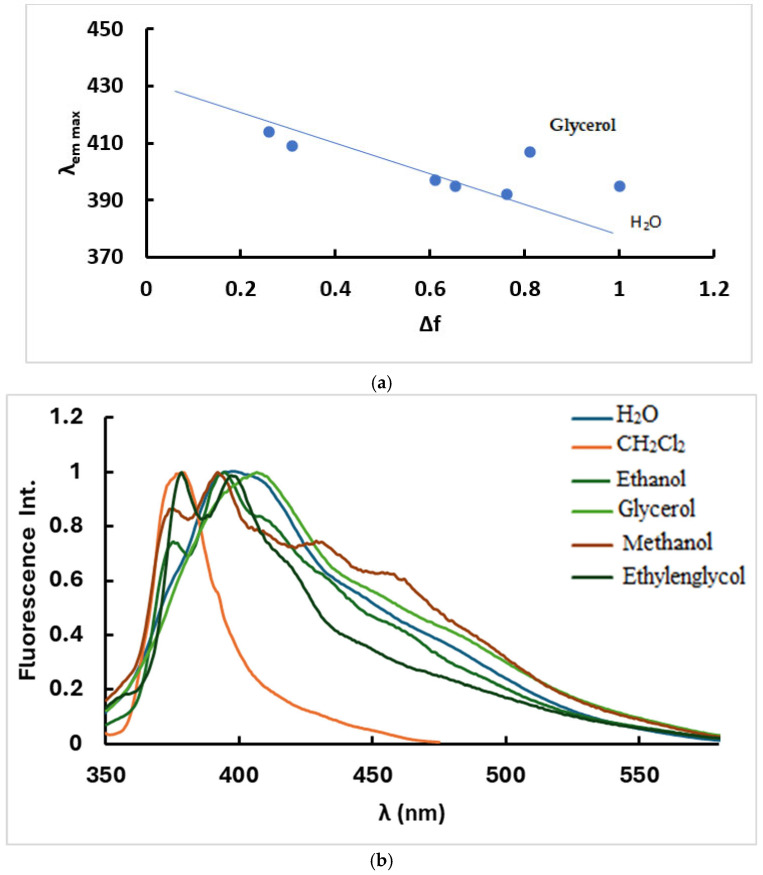
(**a**). Relative polarity effect on emission maxima wavelength of 4 × 10^−5^ M BIMPB, λ_ex_ = λ_abs max_. (**b**). Fluorescence spectra of 4 × 10^−5^ M BIMPB in different solvents, λ_ex_ = λ_ab max_.

Figure 6a,b and Table 1 illustrate the influence of solvent polarity on the fluorescence emission maxima of BIMPB. As solvent polarity increases, the hypsochromic shift (shift to shorter wavelengths) observed in the fluorescence spectra suggests a reduction in the dye’s ground-state dipole moment upon excitation, coupled with an increase in the excited-state dipole moment due to solvent polarization. Notably, water and glycerol exhibit anomalous behavior, deviating from the general trend. This deviation is attributed to their strong hydrogen-bonding interactions, which disrupt the typical solvent polarity-dependent response [56,57,58].

### 3.5. The Combined Effect of Solvent Polarity on Fluorescence Intensity

**Figure 7:**
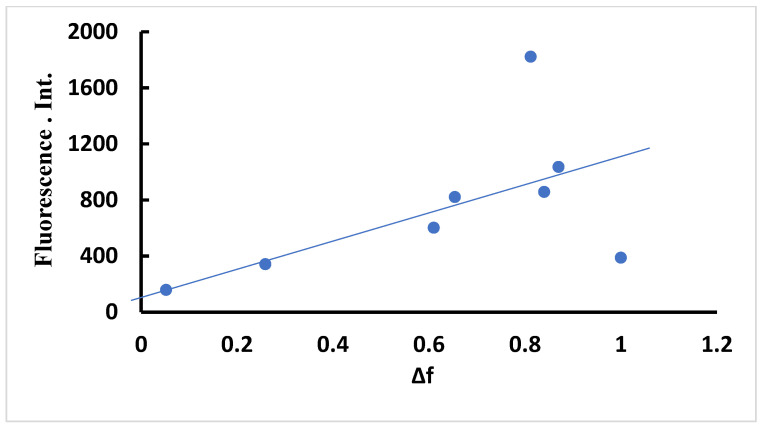
Plot of fluorescence intensity of 4 × 10^−5^ M BIMPB agonist solvent relative polarity (Δf).

Due to the increase in solvent polarity, the non-radiative decay pathways of the dye BIMPB (e.g., internal conversion or vibrational relaxation) are reduced, which enhances the fluorescence quantum yield. This occurs because the energy gap between the ground and excited states increases, as the ground state (more polar) is stabilized more significantly compared to the excited state (less polar). Despite the slight stabilization of the excited state by polar solvents, the dominant effect is the amplification of the energy gap resulting from ground-state stabilization, as illustrated in Figure 7.

As illustrated in Figure 5, the emission behavior in glycerol and water deviates from the general polarity trend. While glycerol’s high viscosity could theoretically restrict non-radiative internal molecular motions, the observed spectroscopic profile suggests that specific solute–solvent hydrogen bonding is the governing factor. The hydroxyl groups in both water and glycerol interact directly with the BIMPB core, disrupting the standard polarity-based stabilization and facilitating non-radiative decay or red-shifted emissive states. The fact that water (low viscosity) and glycerol (high viscosity) induce analogous deviations underscores the observation that the photophysical response is sensitive to the chemical nature of the hydrogen-bonding network rather than the mechanical resistance of the medium (water shows greater hydrogen bonding than glycerol).

**Figure 8:**
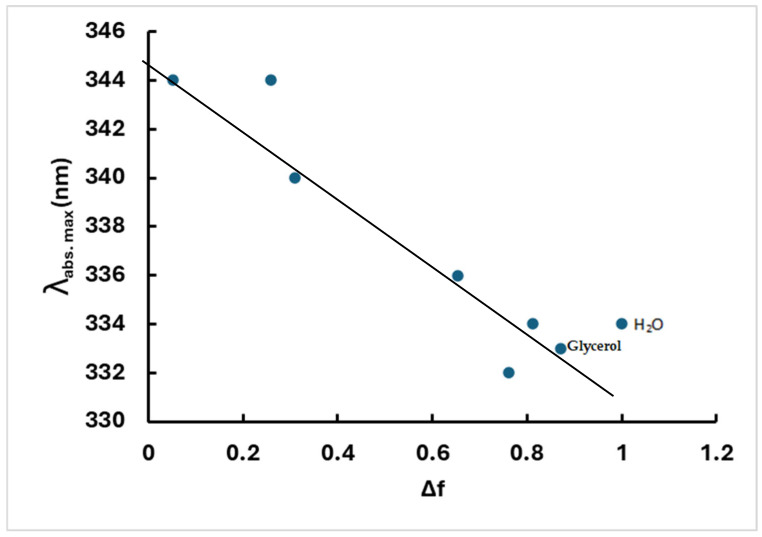
Plot of absorption maximum wavelengths of 4 × 10^−5^ M BIMPB against solvent relative polarity.

At room temperature and a concentration of 4 × 10^−5^ M, the UV/Vis absorption spectra of the BIMPB dye were obtained in several solvents with different polarities. Figure 8 demonstrates how solvent polarity affects where the electronic absorption maxima are located. As the solvent polarity of the medium increases, hypsochromic band shifts in the electronic absorption spectra of BIMPB are observed and reflect a decrease in the dye molecule’s ground-state dipole moment upon excitation and an increase in its ground-state dipole moment as a result of solvent polarization [57,58,59].

**Figure 9:**
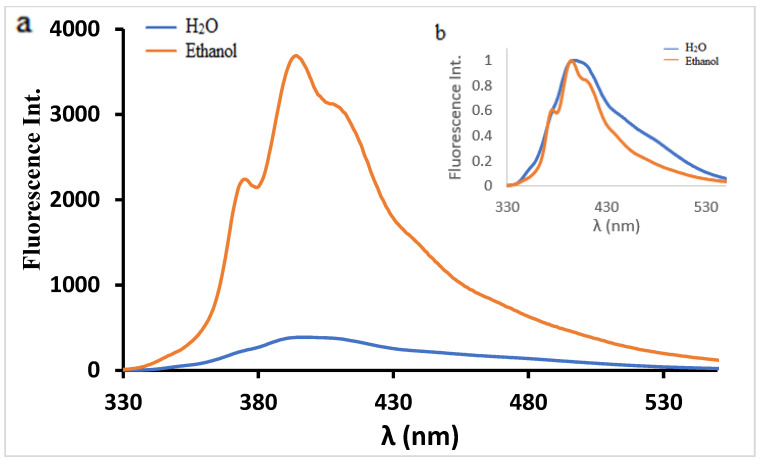
(**a**): Fluorescence spectra of 4 × 10^−5^ M BIMPB in H_2_O and ethanol. (**b**): Normalized fluorescence spectra of 4 × 10^−5^ M BIMPB in H_2_O and ethanol, λ_ex_ = 314 nm.

Figure 9a,b illustrate the reduced fluorescence intensity of BIMPB in water compared to ethanol, which is attributed to hydrogen-bonding effects. In water, strong hydrogen bonds stabilize the excited state of the molecules, decreasing the likelihood of fluorescence emission. In contrast, ethanol has weaker hydrogen bonds, allowing for more effective emission properties (see Table 1). Glycerol’s chemical structure allows it to balance the effects of hydrogen bonding and polarity, leading to enhanced fluorescence emission compared to water and ethanol (due to reduced quenching, taking into account its high viscosity). This aligns with the solvent parameters in Table 1 and highlights the interplay between solvent properties and excited-state dynamics.

### 3.6. Concentration Quenching (Self-Quenching)

**Figure 10:**
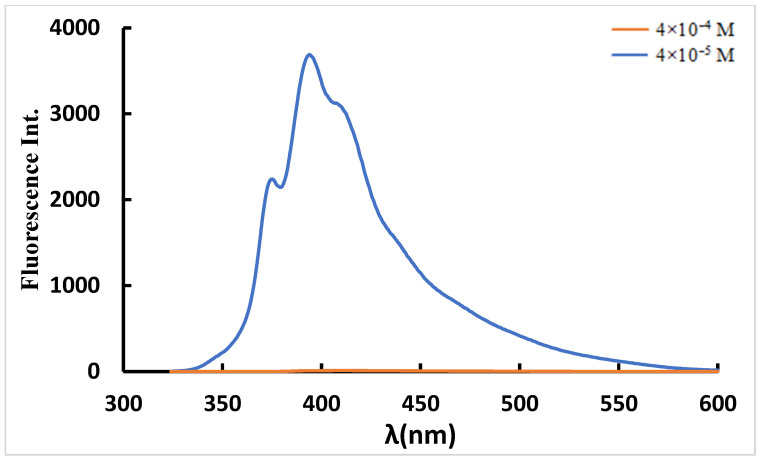
The fluorescence of BIMPB in ethanol at different concentrations, λ_ex_ = 314 nm.

**Figure 11:**
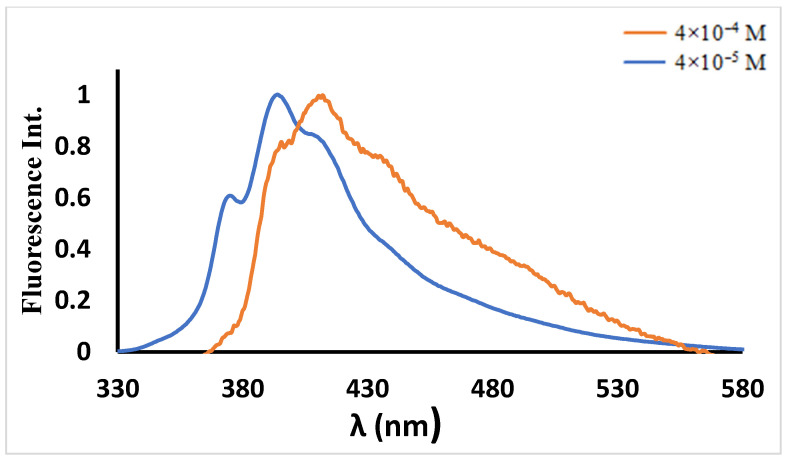
Normalized fluorescence spectra of 5 × 10^−5^ and 4 × 10^−4^ M BIMPB in ethanol, λ_ex_ = 314 nm.

The fluorescence intensity of BIMPB diminishes when the dye concentration is raised to one-tenth of its initial level, as illustrated in Figure 10. This reduction suggests the presence of a quenching phenomenon, likely linked to the reabsorption of excitation photons at higher concentrations, known as the inner filter effect [60]. To mitigate the inner filter effect, it is advisable to keep the dye concentration within a low range [61]. The 18 nm red shift in the fluorescence maximum wavelength at higher concentrations shown in Figure 11 highlights concentration-dependent aggregation behavior, where BIMPB transitions from monomeric emission (shorter wavelength) to aggregated states (longer wavelength). This is critical for applications requiring controlled fluorescence properties (e.g., sensors, optoelectronics) [55].

**Figure 12:**
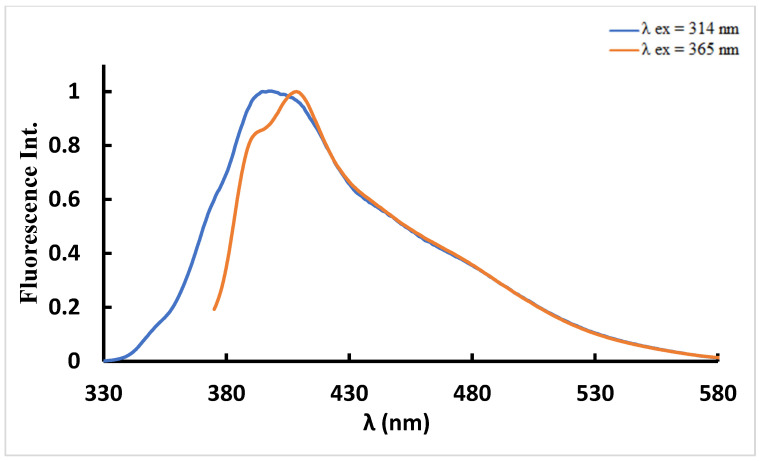
Normalized fluorescence spectra of 4 × 10^−5^ M BIMPB in H_2_O, λ_ex_ = 314 and 365 nm.

The variation in the fluorescence maximum wavelength of BIMPB upon changing the excitation wavelength (Figure 12) suggests a deviation from the expected behavior of a single fluorophore. While Kasha’s Rule dictates that emission typically proceeds from the lowest excited state (S_1_), the observed shifts (396 nm vs. 410 nm) indicate that the 314 nm and 365 nm wavelengths selectively excite different ground-state populations. These likely correspond to different tautomeric isomers or hydrogen-bonded complexes stabilized by the aqueous environment. This heterogeneity in the ground state allows for the targeting of specific electronic species, each undergoing its own S_1_ to S_0_ relaxation pathway, consistent with the fundamental principles of excited-state relaxation.

### 3.7. Effect of Medium Acidity

**Figure 13:**
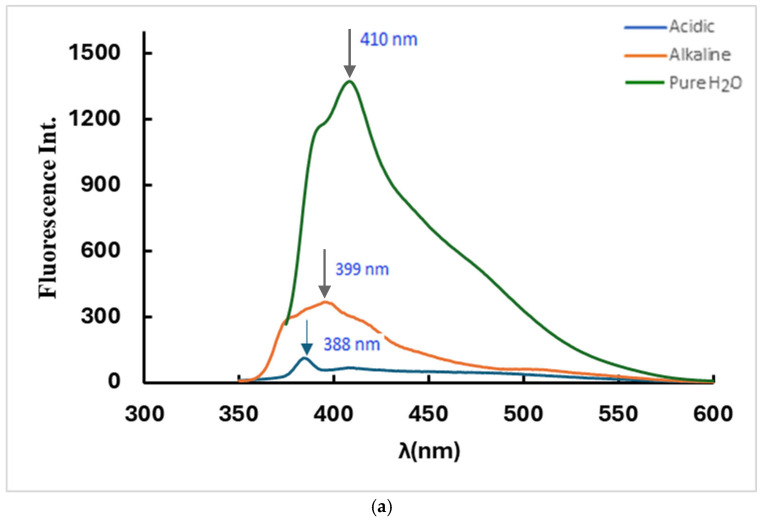

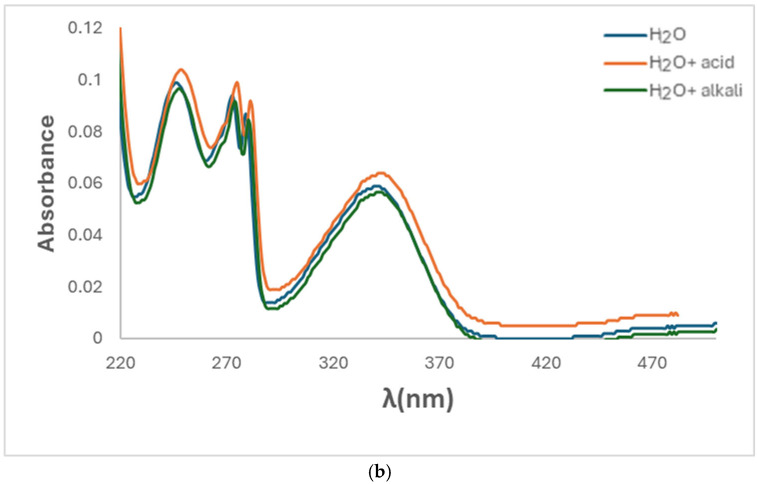
(**a**). Absorption spectra of 4 × 10^−5^ M BIMPB in pure H_2_O, acidic and alkaline solution, λ_ex_ = 350 nm. (**b**). Absorption spectra of 4 × 10^−5^ M BIMPB in pure H_2_O and in aqueous acidic and alkali solution.

As shown in the table below, at 410 nm, a stable, highly conjugated electronic structure that supports efficient radiative decay is reflected. Upon shifting to alkaline conditions (pH = 9, adjusted with NaOH), the emission undergoes an 11 nm blue shift to 399 nm, accompanied by significant fluorescence quenching—likely due to deprotonation or altered dipole moment, which raises the energy of the S_1_ → S_0_ transition. In acidic medium (pH = 3, adjusted with HCl), an even more pronounced 23 nm blue shift to 388 nm occurs, along with near-complete fluorescence suppression [62,63]. This is attributed to the protonation of the benzimidazole or imine nitrogen atoms, which disrupts π-conjugation and promotes non-radiative decay pathways. As a result, BIMPB demonstrates exceptional pH-dependent fluorescence response, particularly in acidic environments, positioning it as a highly sensitive fluorescent probe. To further assess the stability of BIMPB under varying pH conditions, we systematically recorded its UV–Vis absorption spectra across the entire studied pH range (Figure 13b). The absorption profiles in both acidic and alkaline media were found to be virtually indistinguishable from that in neutral solution, with no detectable shifts in λ_ab max_, changes in molar absorptivity, or appearance of new absorption bands that could indicate degradation. This spectral invariance strongly confirms the chemical stability of BIMPB over the tested pH range and rules out hydrolysis or structural decomposition under the experimental conditions employed. These characteristics underscore its strong potential for real-time intracellular pH sensing, biological imaging, and the development of specialized chemical sensors for monitoring acidic conditions in industrial or environmental settings.

### 3.8. Metal Ion Sensor

**Figure 14:**
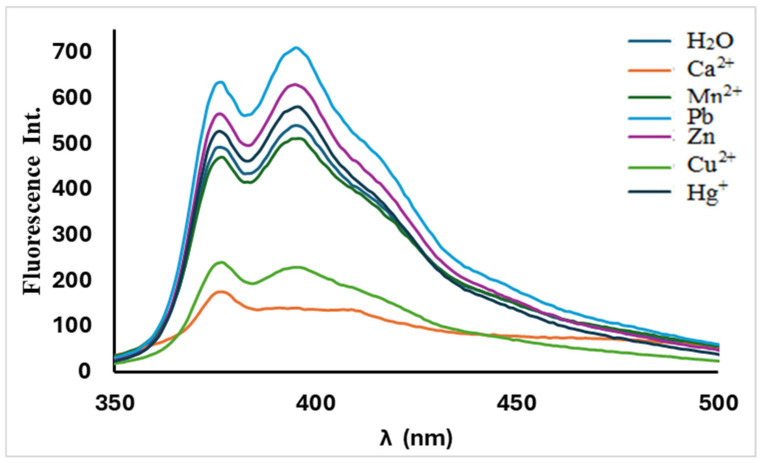
Fluorescence spectra of 4 × 10^−5^ M BIMPB in buffered aqueous solution using Trizma with six different metal ions [M^n+^] = 8 × 10^−5^ M, λ_ex_ = 350 nm.

The fluorescence intensity of BIMPB exhibited distinct responses to the addition of various metal ions. Trace amounts of mercury, lead, nickel, and manganese ions caused only a slight decrease in fluorescence, whereas calcium (Ca^2+^) and copper (Cu^2+^) ions significantly reduced the fluorescence intensity to less than one-third of its original value in water, as shown in Figure 14. This marked reduction is attributed to the strong paramagnetic properties of Cu^2+^, which quench fluorescence by interfering with the excited states through mechanisms such as electron or energy transfer. For Ca^2+^, the observed quenching at low concentrations likely arises from the formation of coordination complexes with the molecule, altering its electronic structure, stabilizing the excited state, and reducing emission efficiency.

**Figure 15:**
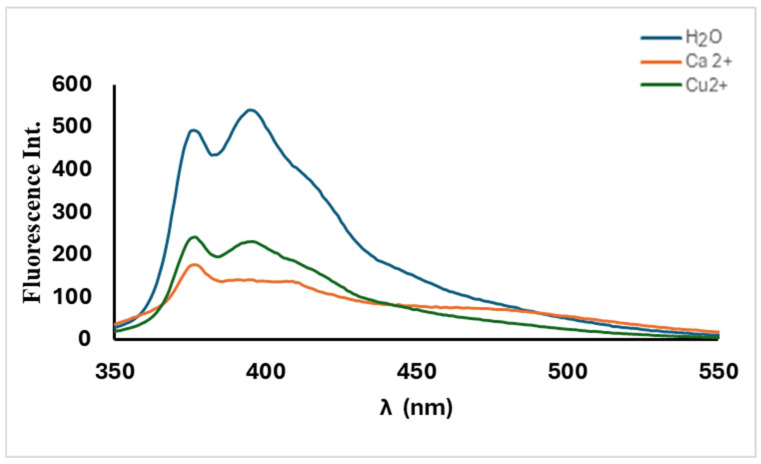
Fluorescence spectra of 4 × 10^−5^ M BIMPB in buffered aqueous solution using Trizma and with Ca and Cu ions [M^n+^] = 8 × 10^−5^ M, λ_ex_ = 350 nm.

In the fluorescence spectra, a notable inversion in peak heights was observed upon adding metal ions. In water, the emission intensity peaked at 397 nm, but with the addition of Cu^2+^ and Ca^2+^, the intensity at 378 nm surpassed that at 397 nm, indicating a blue shift. This shift is attributed to the preferential formation of complexes with specific molecular states, redistributing energy among excited states and modifying the local polar environment around the molecule (Figure 15).

**Figure 16:**
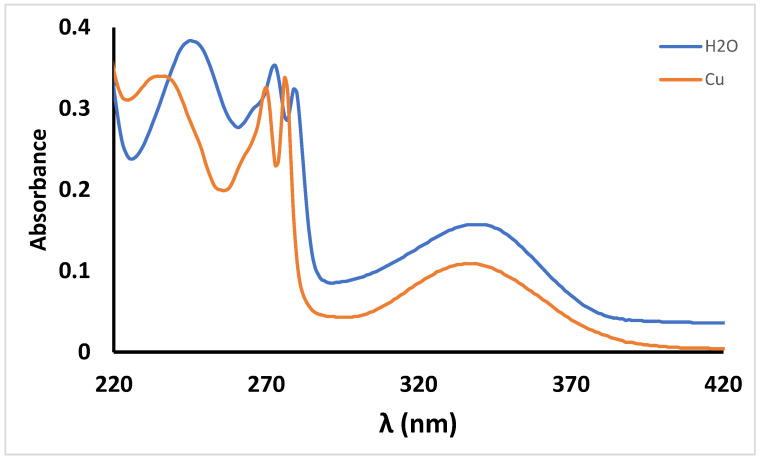
Absorption spectra of 4 × 10^−5^ M BIMPB in buffered aqueous solution using Trizma and in 8 × 10^−5^ M Cu^2+^.

The absorption spectra shown in Figure 16 further revealed significant shifts in high-energy peaks (277 nm, 271 nm, 245 nm), corresponding to π → π* transitions within aromatic rings. These shifts are indicative of the formation of coordination complexes between Cu^2+^ and BIMPB, altering the energy levels of the molecular orbitals involved in these transitions. Conversely, the absorption peak at 340 nm (S_0_ → S_1_ transition) remained stable, suggesting that this lower-energy n → π* transition is less sensitive to perturbations caused by Cu^2+^ binding.

The similarity in fluorescence quenching induced by Ca^2+^ and Cu^2+^ shown in Figure 14 highlights their interaction with the excited states of BIMPB. While Ca^2+^ induces subtle structural changes or coordination complex formation affecting radiative decay pathways, Cu^2+^ introduces additional quenching mechanisms due to its paramagnetic nature. The sensitivity of fluorescence to minor environmental changes underscores the potential of BIMPB as a selective fluorescent sensor for Cu^2+^ and Ca^2+^ ions. The sensing mechanism of BIMPB involves two distinct electronic pathways. For Cu^2+^, its paramagnetic nature and unfilled d-orbitals facilitate Photoinduced Electron Transfer (PET) and Intersystem Crossing (ISC), diverting the excited-state population to non-radiative triplet states. Conversely, Ca^2+^ detection is governed by Coordination-Induced Electronic Perturbation. The binding of Ca^2+^ stabilizes the ground state (S_0_) more effectively than the excited state (S_1_), widening the energy gap and causing the observed hypsochromic (blue) shift. Additionally, the resulting molecular rigidity enhances non-radiative decay through increased coupling with the solvent environment, leading to the observed quenching.

### 3.9. DNA Binding Studies

#### 3.9.1. UV-Vis Spectral Study

**Figure 17:**
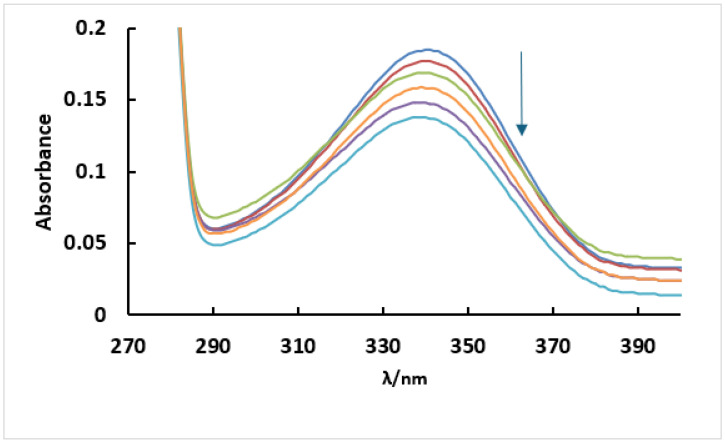
UV–Vis absorption spectra of BIMPB (4 × 10^−5^ M) in the absence and presence of DNA. The concentrations of DNA were (1) 0.0, (2) 2.5 × 10^−6^ M, (3) 5.0 × 10^−6^ M, (4) 7.5 × 10^−6^ M, (5) 1.0 × 10^−5^ M, and (6) 1.25 × 10^−5^ M.

The interaction between compound BIMPB (4 × 10^−5^ M) and DNA at different concentrations is depicted by the UV-Vis absorption spectra in Figure 17. When DNA is not present, BIMPB has a clear absorption peak at 342 nm (curve 1). The addition of DNA causes a discernible hypsochromic shift (blue shift), with the absorption peak moving from 342 nm to 340 nm at concentrations ranging from 2.5 × 10^−6^ M to 1.25 × 10^−5^ M (curves 2–6). Furthermore, the presence of DNA resulted in a drop in absorbance (hypochromic effect), suggesting that BIMPB absorption intensity decreased when it was bound to DNA. When combined with the blue shift, this hypochromic effect indicates a substantial interaction between BIMPB and DNA, most likely because of electrostatic binding or intercalation, which modifies BIMPB’s electrical environment [29].

To quantify the binding affinity between BIMPB and DNA, the binding constant (*K_b_*) was determined using the following equation [60]:

(2) $$\frac{Ao}{Ao-A}=\frac{\varepsilon_{G}}{\varepsilon_{H-G}-\varepsilon_{G}}+\frac{\varepsilon_{G}}{\varepsilon_{H-G}-\varepsilon_{G}}\times \frac{1}{k_{b}}\frac{1}{C_{DNA}}$$

**Figure 18:**
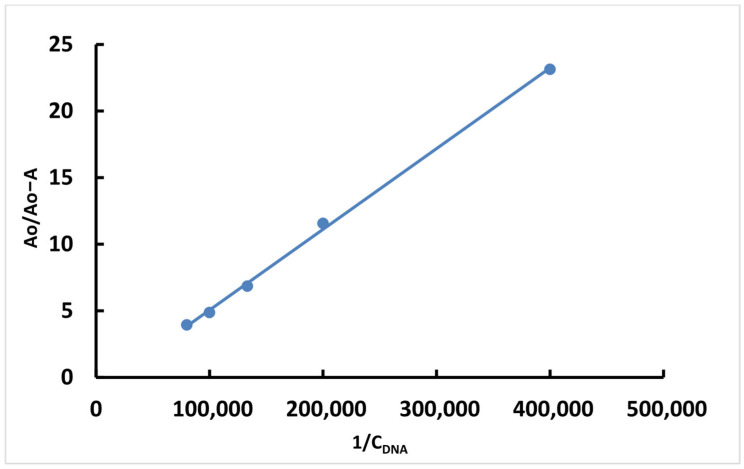
Plot of log (Ao/Ao-A) versus log 1/C_DNA_.

The absorption spectra at λ_max_ for the free BIMPB and the BIMPB B-DNA complex are A_O_ and A, respectively. The absorption coefficients for the guest BIMPB and the complex BIMPB-DNA are ε_G_ and ε_H-G_, respectively. The binding constant *K_b_* was determined using a plot of A_O_/A_O_-A vs. 1/C_DNA_ (Figure 18). The significant shift and hypochromic effect indicate a strong interaction between BIMPB and DNA, confirmed by the high binding constant (*K_b_* = 2.1 × 10^5^ M^−1^). This value indicates the establishment of a stable BIMPB-DNA complex and emphasizes the strong binding affinity of the two molecules.

#### 3.9.2. Fluorescence Studies

**Figure 19:**
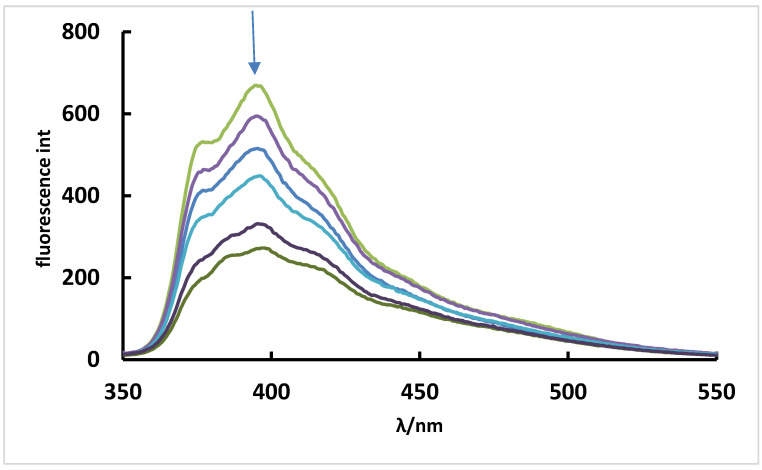
Fluorescence spectra of BIMPB (4 × 10^−5^ M) in the absence and presence of DNA, C_DNA_ = (1) 0.00, (2) 3.0 × 10^−5^, (3) 6.0 × 10^−5^, (4) 9.0 × 10^−5^, (5) 1.2 × 10^−4^, (6) 1.5 × 10^−4^ M.

Fluorescence spectroscopy is a useful and exact method for measuring the interaction and binding ratios between BIMPB and DNA [64]. In this work, the fluorescent characteristics of BIMPB were studied to see how it interacts with DNA. Unlike DNA, which does not develop spontaneously, BIMPB fluoresces brightly in aqueous conditions. When exposed to a certain wavelength of light, BIMPB exhibits a characteristic fluorescence pattern. Figure 19 displays the fluorescence spectra of BIMPB (4 × 10^−5^ M) with and without DNA, at concentrations ranging from 0.0 to 1.5 × 10^−4^ M. The results show that when the concentration of DNA increases, the fluorescence intensity of BIMPB is reduced dramatically, reaching 58.9% at the maximum concentration. This significant reduction in fluorescence, together with a slightly weakened emission wavelength, clearly shows that the interaction between BIMPB and DNA happens via an intercalation process [65]. The significant drop in fluorescence intensity suggests that the BIMPB excited state is substantially disrupted when bound to DNA, which supports the establishment of a stable BIMPB-DNA complex.

**Figure 20:**
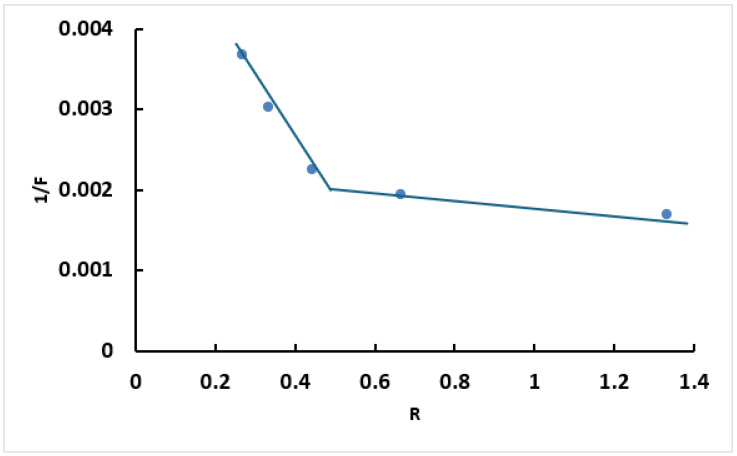
Plot of 1/F versus R for BIMPB with DNA.

Figure 20 shows a graph showing the relationship between 1/F and R, where F is the fluorescence intensity, and R is the ratio of DNA concentration to BIMPB concentration, to determine the interaction ratio. The binding ratio of 1:0.4 between BIMPB and DNA is confirmed by the linear inflection point shown in this figure.

**Figure 21:**
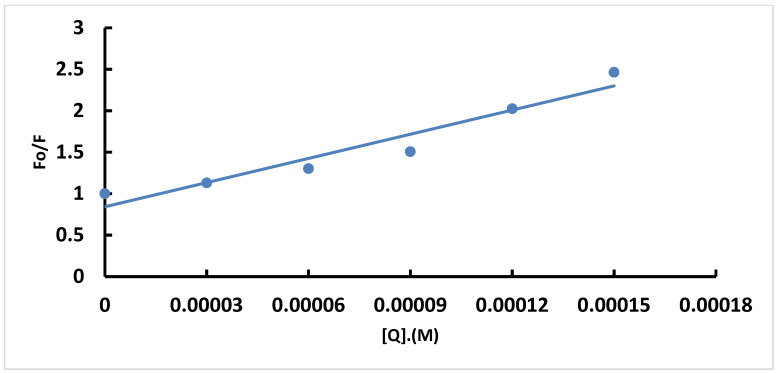
Plot of Fo/F versus [Q] for BIMPB with DNA.

Quenching efficiency was quantified using the Stern–Volmer equation [20]:

(3) $$\frac{F_{o}}{F}=1+k_{q}\tau_{o}\left[Q\right]=1+k_{sv}\left[Q\right]$$

where K_sv_ is the Stern–Volmer constant, and [Q] is the DNA concentration. A linear plot of Fo/F versus [Q] confirmed Stern–Volmer behavior (Figure 21), with K_sv_ determined as 9.72 × 10^3^ M^−1^. Using the formula K_sv_/$\tau_{o}$(where $\tau_{o}$ = 1 × 10^−8^ s), the bimolecular quenching rate constant, k_q_, was determined to be 9.72 × 10^11^ M^−1^ s^−1^. This value is much higher than the usual diffusion-controlled limit (10^10^ M^−1^ s^−1^), suggesting that the quenching process is static [66,67], most likely because of BIMPB and DNA forming a non-fluorescent ground-state complex.

**Figure 22:**
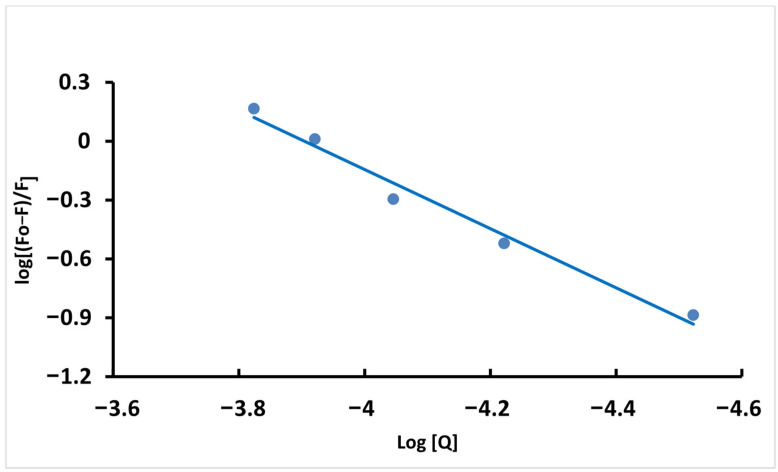
The Stern–Volmer plot illustrates the fluorescence quenching of BIMPB in the presence of DNA.

The binding constant (K_b_) and number of binding sites (n) were determined according to the following relationship [68], as shown in Figure 22:

(4) $$\log\frac{F_{o}-F}{F}=\log k_{b}+n\log\left[Q\right]$$

where F and Fo are the fluorescence intensities of the BIMPB-DNA complex and free fluorophore, respectively, and [Q] is the DNA concentration. The y-intercept yields log *K*_b_, while the slope corresponds to n. In contrast to weak (<10^4^ M^−1^) or strong (>10^6^ M^−1^) interactions, the data (*K*_b_ = 2.52 × 10^5^ M^−1^; n = 1.507) imply a moderately strong interaction, as indicated by the binding constants in the 10^5^–10^6^ M^−1^ range [69].

### 3.10. Molecular Docking

**Figure 23:**
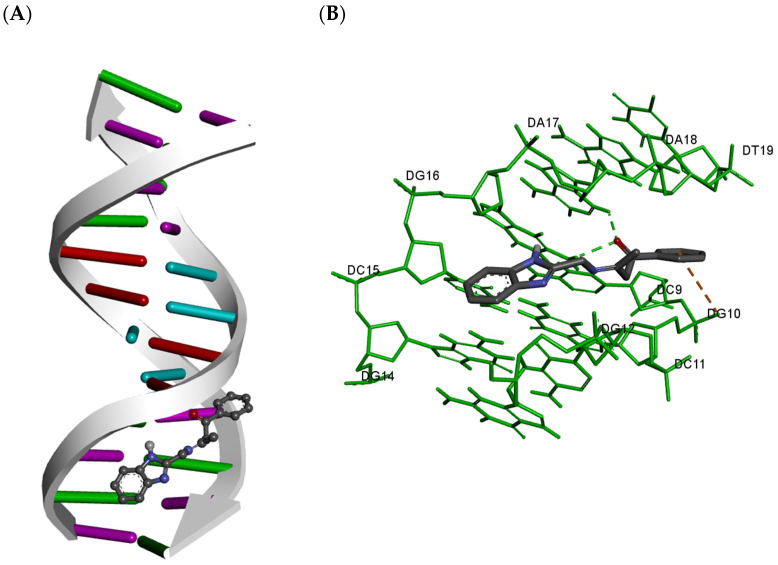
(**A**) The best molecular binding conformation of BIMPB with 1BNA. (**B**) Non-covalent interactions of the BIMPB compound with 1BNA.

Molecular docking simulations were conducted to explore the binding interactions between compound BIMPB and B-DNA (PDB ID: 1BNA). This computational approach is critical for identifying optimal binding sites and elucidating non-covalent interactions, providing valuable insights for drug design and molecular recognition studies. The docking results, presented in Figure 23, demonstrate that BIMPB preferentially binds to specific DNA sequences, forming a stable complex. Key interactions stabilizing the BIMPB-DNA complex include conventional hydrogen bonds and π–anion and π–sigma interactions. The calculated binding affinity of −8.3 kcal/mol reflects a robust interaction. Notably, hydrogen bonds were identified with DA17 (2.02 Å) and DG16 (2.38 Å), alongside a π–anion interaction with DG10 (4.39 Å). These computational findings align with experimental evidence of strong BIMPB-DNA binding, as indicated by the high binding constant derived from UV-Vis spectral studies. The docking-identified interactions, particularly hydrogen bonds and π–anion contacts, likely contribute to the experimentally observed binding affinity, validating the computational model. While docking provides a static view of molecular interactions, this synergy with experimental data enhances confidence in the predicted binding mode and underscores BIMPB’s potential as a DNA-targeting molecule for therapeutic applications.

### 3.11. Theoretical Study

**Figure 24:**
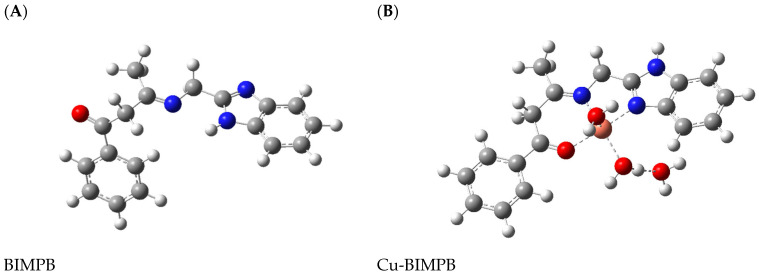
Model structures for (**A**) BIMPB and (**B**) Cu-BIMPB.

**Figure 25:**
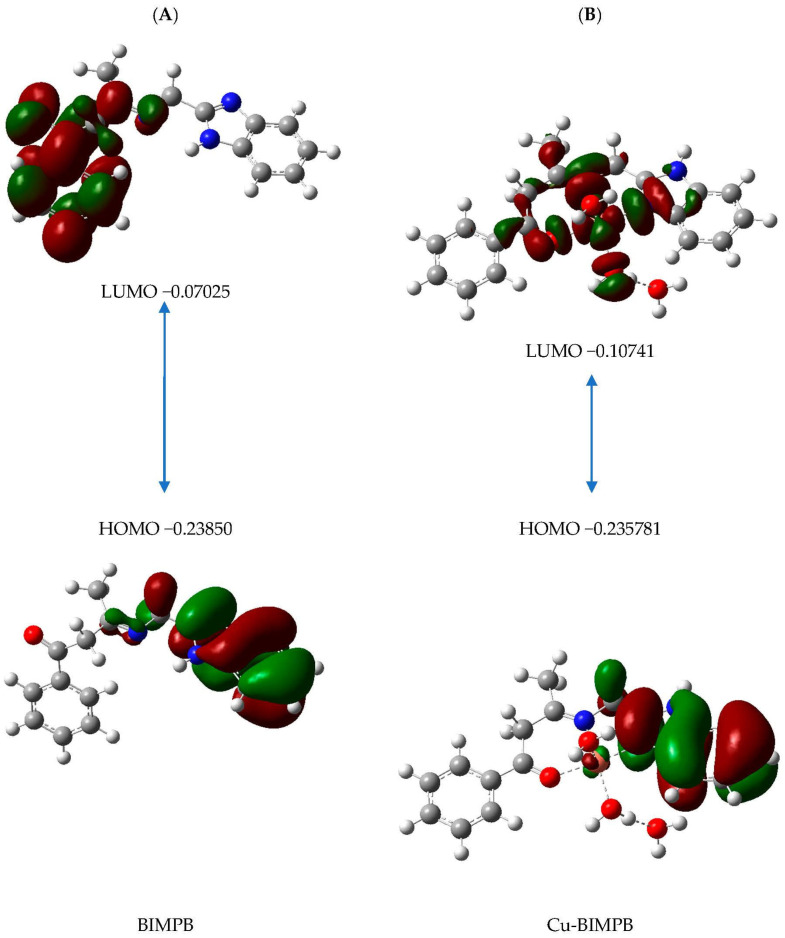
Frontier orbitals and their energies for (**A**) BIMPB and (**B**) Cu-BIMPB.

Computational studies were conducted using the Gaussian 09 software suite, using Density Functional Theory (DFT) and Time-Dependent DFT (TDDFT) methodologies to elucidate the structural and electronic properties of the Cu^2+^-BIMPB complex and their impact on fluorescence behavior [70]. The molecular geometry of BIMPB and its copper complex (Cu-BIMPB) was optimized using the PBE1PBE functional, with the 6-31G(d) basis set applied to non-metal atoms and the LANL2DZ basis set used for the copper atom. Vibrational frequency analyses at the same level confirmed the absence of imaginary frequencies, verifying the stability of the optimized structures. To account for the aqueous experimental conditions, TDDFT calculations employed the SMD solvation model at the PBE1PBE/6-31+G(d)/LANL2DZ level. The optimized structure of BIMPB is shown in Figure 24A, while the Cu-BIMPB complex is depicted in Figure 24B. In the complex, the Cu^2+^ ion adopts a five-coordinate geometry, binding to BIMPB via two nitrogen atoms and one oxygen atom, with additional coordination to two water molecules. The calculated bond distances are: 1.96 Å and 2.02 Å for Cu–N, 1.95 Å for Cu–O, and 2.00 Å and 2.24 Å for Cu–H_2_O. These bond lengths highlight the effective coordination of Cu^2+^ by BIMPB’s functional groups. TDDFT calculations provided insights into the electronic transitions of BIMPB and Cu-BIMPB, with frontier molecular orbitals (FMOs) being illustrated in Figure 25. Key transition data, including wavelengths and oscillator strengths, are summarized in Table 2. For BIMPB, the electron density in the HOMO and LUMO is predominantly localized on the aromatic rings. In contrast, the Cu-BIMPB complex exhibits intramolecular charge transfer (ICT), with the HOMO electron density concentrated on the fused aromatic rings and the LUMO distributed across the copper center and ligand atoms bonded to it. The reduced oscillator strength in the Cu-BIMPB complex (Table 2) correlates with fluorescence quenching, likely due to Cu^2+^ coordination, which alters the electronic structure and suppresses radiative transitions. The HOMO and LUMO energies of the complex were calculated to be −6.42 eV and −2.92 eV, respectively. The HOMO and LUMO energies for the free ligand in water are −6.49 eV and −1.91 eV. Although the quantum yield cannot be accessed directly via DFT calculations, the HOMO–LUMO energy difference suggests that the quantum yield of the complex is smaller than that of the free ligand, which is experimentally 0.04 in water.

We performed additional TD-DFT calculations on the Ca^2+^-BIMPB complex. As shown in Table 2, the oscillator strength of the S_1_ state decreases dramatically upon complexation with both Cu^2+^ (f = 0.0003) and Ca^2+^ (f = 0.0002), compared to the free ligand (f = 0.0074). The even lower value for the Ca^2+^ complex is consistent with the experimentally observed stronger fluorescence quenching in the presence of Ca^2+^.

## 4. Conclusions

The study of BIMPB’s photophysical properties reveals a dynamic interaction between electronic transitions, solvent effects, and molecular aggregation. The strong absorbance at 212 nm is mainly due to a spin-allowed S_0_ → S_2_ transition, while weaker features like the 304 nm shoulder in water arise from S_0_ → S_1_ transitions. A large Stokes shift (1.43 × 10^5^ cm^−1^) in CH_2_Cl_2_ indicates efficient energy dissipation and high fluorescence efficiency. Emission properties are highly solvent-dependent; polarity-induced shifts and hydrogen bonding (in water and glycerol) affect emission intensity and wavelength, often causing red shifts or quenching. At higher concentrations, BIMPB exhibits aggregation-induced red shifts (~18 nm) and self-quenching, reflecting sensitivity to environmental changes. Fluorescence also varies with excitation wavelength, indicating distinct π → π* and n → π* transitions. Notably, pH-dependent emission shifts (11–23 nm) suggest BIMPB functions as a pH-sensitive fluorescent probe. Spectroscopic analysis shows that BIMPB binds effectively with DNA, primarily through intercalation. Both UV–Vis and fluorescence spectroscopy confirm this interaction, as evidenced by hypochromic effects and fluorescence quenching upon DNA addition. The binding constant (2.1 × 10^5^ M^−1^) indicates moderate affinity, supporting its suitability for biosensing and therapeutic applications. These insights into BIMPB’s behavior provide a foundation for the further development of benzimidazole-based compounds in molecular sensing, optoelectronics, and anticancer strategies.

## Figures and Tables

**Table 1:** Fluorescence maximum (λ_em max_) and absorption maximum wavelengths (λ_abs_,_max_), photochemical quantum yield (ϕ_c_), fluorescence quantum yield (ϕ_f_) and Stokes shift values (measured values) for BIMPB in various solvents with different dielectric constants and relative polarities.

Solvents	λ_abs max_ (nm)	λ_em max_ (nm)	ε LM^−1^ cm^−1^	Δf	Dielectric Constant	ϕ_c_	ϕ_f_	Stokes Shift. 10^5^ cm^−1^	Flu. Int.
CCl_4_	344	501	1750	0.052	2.24	0.05	0.01	0.637	159
CHCl_3_	344	414	1275	0.259	4.81	0.06	0.04	1.43	342
CH_2_Cl_2_	340	409	2025	0.309	8.93	0.05	0.32	1.45	1124
EtOH	336	395	8650	0.654	24.5	0.06	0.06	1.69	821
CH_3_OH	332	392	2075	0.762	32.7	0.04	0.03	1.67	454
DMSO	333	398	1925	0.87	20.7	-	0.26	1.54	1036
Ethylene glycol	345	397	1475	0.61	41	-	0.02	1.92	602
Glycerol	334	407	1550	0.812	46.5	0.03	0.46	1.37	1822
H_2_O	334	395	1350	1	80.1	0.04	0.04	1.64	388
n-Butanol	339	395	2000	0.84	17.8	0.04	0.06	1.79	858

**Table 2:** TDDFT outcomes of certain transitions, including associated absorbance energies and oscillator strengths.

Complex	Transition	λ (nm)	Oscillator Strength
BIMPB	HOMO > LUMO	319	0.0074
Cu-BIMPB	HOMO(b) > LUMO(b)	388	0.0003
Ca-BIMPB	HOMO > LUMO	311	0.0002

## Data Availability

The original contributions presented in this study are included in the article. Further inquiries can be directed to the corresponding author.
